# Bioinspired micro‐ and nanostructured systems for cancer therapy

**DOI:** 10.1002/mco2.70025

**Published:** 2024-11-28

**Authors:** Rui Yang, Bing Zhang, Xiawei Fei, Shanshan Cong, Shaojie Zhao, Tao Zhou, Yanting Shen

**Affiliations:** ^1^ Department of Research Institute for Reproductive Medicine and Genetic Diseases, Wuxi Maternity and Child Health Care Hospital Jiangnan University Wuxi China; ^2^ Department of Gynaecology Wuxi Maternity and Child Health Care Hospital, Jiangnan University Wuxi China; ^3^ Department of Urology Qingpu Branch of Zhongshan Hospital Affiliated to Fudan University Shanghai China; ^4^ Department of Urology and Andrology Gongli Hospital of Shanghai PudongNew Area Shanghai China; ^5^ Department of Laboratory for Research in Urology and Andrology Gongli Hospital of Shanghai PudongNew Area Shanghai China

**Keywords:** biocompatibility, bioinspired micro‐ and nanostructured systems, cancer therapy, traverse physiological barriers

## Abstract

Numerous organisms in nature have demonstrated enhanced biocompatibility, precise tumor targeting capabilities, and efficient tissue traversal within the human body. Drawing inspiration from these organisms, researchers have employed bioengineering, bioconjugation, and micro‐ or nanotechnology to fabricate bioinspired micro‐ and nanostructured systems. These systems play a crucial role in addressing the limitations of conventional anticancer drugs and nanomaterials concerning biocompatibility, effective penetration of physiological barriers, as well as selective tumor targeting, thereby leading to improved therapeutic efficacy while minimizing nonspecific adverse effects on healthy cells. Consequently, extensive exploration of these bioinspired micro‐ and nanostructured systems has been undertaken across various cancer treatment modalities with some progressing into preclinical or clinical stages. However, our understanding of this field remains limited which may impede research progress, clinical translation efforts, and practical applications. Therefore, this study presents a systematic classification of bioinspired micro‐ and nanostructured systems for cancer therapy that comprehensively elucidates their sources of inspiration and design principles. Furthermore, it extensively discusses the current status of clinical translation efforts while identifying prevailing challenges and exploring future prospects. This work will establish a robust theoretical framework and serve as a valuable reference to facilitate advancements in research and clinical application within this field.

## 1 INTRODUCTION

In the context of the 21st century, cancer has emerged as a significant public health and socio‐economic concern. The report titled “Global Cancer Statistics 2022” unveiled that there were nearly 20 million new cancer cases worldwide (estimated to reach 35 million by 2050), resulting in approximately 10 million deaths related to cancer.^1^ Despite substantial efforts and improved patient survival rates achieved through conventional therapeutic approaches such as surgery, chemotherapy, and radiation therapy, their efficacy remains limited when dealing with metastatic cancers.^2^ In recent years, with an increasing understanding of the intricate networks between the immune system and the complex tumor microenvironment (TME), strategies aimed at educating and activating the immune system to perform its intended antitumor functions have shown significant antitumor potential. These include the administration of vaccines that stimulate dendritic cells, immune checkpoint blockade therapies that enhance T cell functions, as well as cytokines, antibodies, immune modulators, and immune adjuvants targeting T cells, B cells, and natural killer (NK) cells.^3^, ^4^, ^5^, ^6^ Although immunotherapy represents a remarkable breakthrough in advanced cancer treatment, its clinical success is hindered by low response rates among patients. Similar to conventional treatments, immunotherapy often eliminates tumor cells at the expense of sacrificing numerous normal cells, leading to severe and occasionally fatal side effects.^2^ This underscores the pressing need to address formidable challenges pertaining to precision and safety within current cancer therapies.

Microstructured (with diameters in the micrometer range, typically from 1 to 1000 µm) and nanostructured (referring to materials with a single unit size ranging from 1 to 1000 nanometers, typically from 1 to 100 nanometers) systems have offered unique solutions to address current challenges in treatment precision and safety.^7^, ^8^, ^9^, ^10^, ^11^ They are preferred for topical administration routes, including direct targeting of organs, intraperitoneal, intramuscular, or subcutaneous delivery, rather than systemic drug administration.^12^ Consequently, they can effectively mitigate systemic side effects. However, their treatment of deep‐seated solid tumors or hematological malignancies still exhibits certain limitations. Although certain nanostructured systems administered systemically can effectively target solid tumor tissues through enhanced permeability and retention (EPR) effects, thereby reducing adverse effects on normal tissue, as well as partially addressing the limitations associated with microstructured systems.^13^ It is crucial to acknowledge two significant constraints that still require attention. The first one lies in the limited histocompatibility. Taking pegylated liposomal doxorubicin (Doxil/Caelyx) as an example, this nanostructured drug has gained United States Food and Drug Administration (US FDA) approval for its application in various cancer treatments and has demonstrated efficacy in reducing cardiotoxicity. In comparison with conventional doxorubicin, it exhibits a lower incidence of toxic side effects.^9^ However, due to the issue of histocompatibility, the extensive utilization of pegylated nanostructured systems like Doxil over the past decade has resulted in increasing hypersensitivity reactions, which have progressively escalated to life‐threatening levels.^14^ The second one lies in the limited effective penetration of physiological barriers and specific tumor targeting. This limitation may arise from various physiological barriers, primarily including the formation of a protein corona barrier through interactions between nanoparticles and biomolecules in the bloodstream, immune barriers primarily mediated by the mononuclear phagocyte system, permeability barriers resulting from high interstitial hydraulic pressure and dense extracellular matrix (ECM) in tumors, as well as cell uptake barriers posed by cellular and nuclear membranes.^15^, ^16^, ^17^ Therefore, despite the exponential growth of research on “EPR” and “nanomedicine,” significant challenges persist in achieving clinical translation and efficacy enhancement of nanostructured drugs in patients with solid tumors due to the aforementioned limitations, resulting in only 6% of nanomedicine currently successfully transitions from preclinical research to clinical applications.^12^ In summary, there is an urgent need to further enhance biocompatibility, targeting precision, and therapeutic efficacy of micro‐ and nanomaterials for cancer clinical treatment purposes particularly for solid tumors.

**FIGURE 1:**
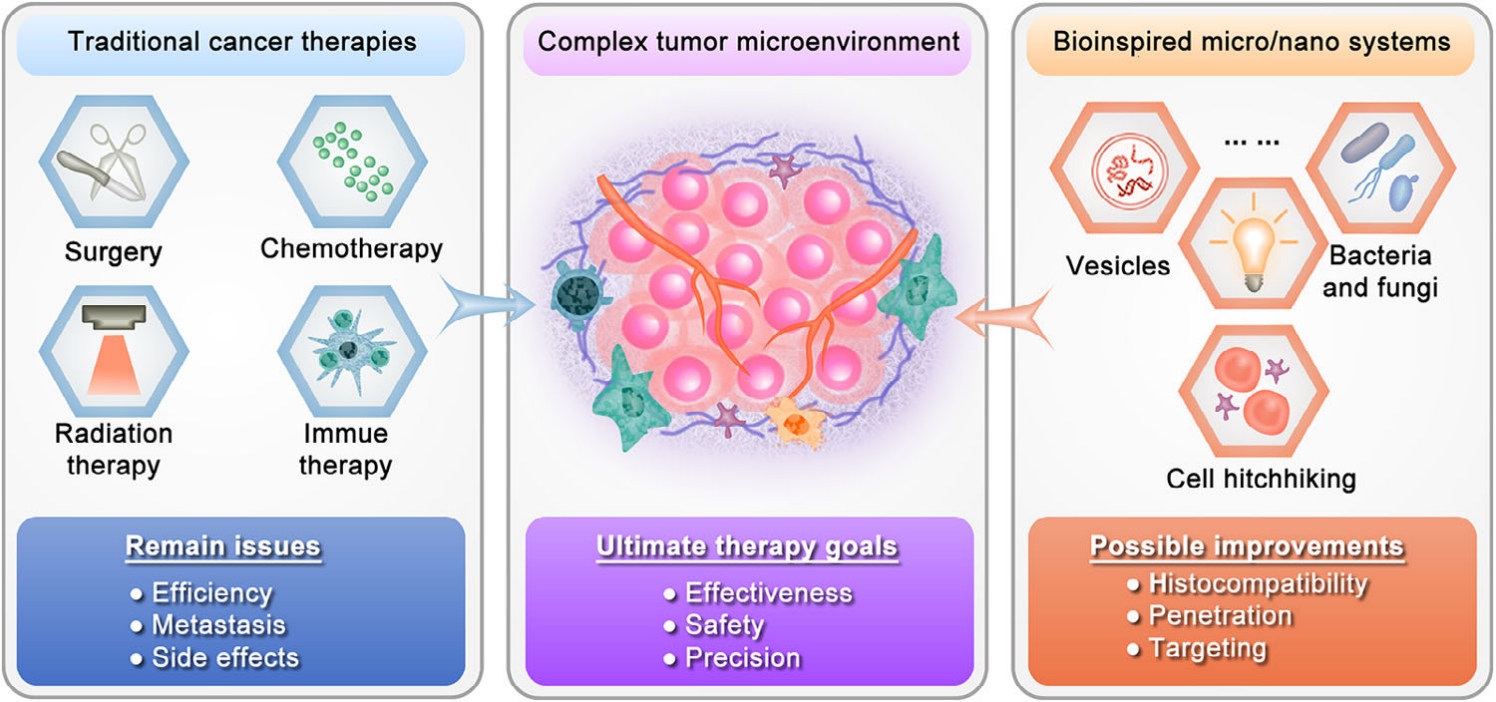
A global comparison between traditional and bioinspired cancer therapies. The limitations of traditional cancer therapies include lack of efficiency to metastatic cancers and the existence of side effects. Bioinspired micro/nano systems may have better improvements on the aspects of histocompatibility, penetration, and targeting.

Bioinspired micro‐ and nanostructured systems hold great potential in addressing the limited histocompatibility, enhancing effective penetration of physiological barriers, and enabling specific tumor targeting (Figure 1). Bioinspired micro‐ and nanostructured systems refer to constructs that are designed or created by drawing inspiration from biological structures, functions, or processes found in nature, and drug delivery systems inspired by biology operate by mimicking aspects such as structure, shape, locomotion, appearance, and surface characteristics.^18^ Unlike bio‐derived systems that are exclusively composed of biomolecules and remain unaltered, bioinspired systems have been subjected to artificial modifications. Consequently, some studies have included micro‐ and nanostructured bio‐derived systems within the category of bioinspired micro‐ and nanostructured systems.^19^ In this review, we follow suit. Since the inception of bionics in the 1960s, bioinspired materials science has emerged as a pivotal research field addressing various challenges. In recent years, advancements in comprehending the interactions between naturally occurring bioactive substances or biological processes found in plants, microorganisms, and animals with the human body, cellular systems, and TME have garnered increased attention toward bioinspired micro‐ and nanostructured systems for surmounting limitations associated with biocompatibility, penetration through biological barriers, and targeted delivery capabilities of conventional micro‐ and nanostructured systems.^18^ For instance, leveraging techniques such as bioengineering, bioconjugation, and micro‐ or nanotechnology enables the development of tumor‐specific colonized microorganisms and endogenous cell membrane‐inspired micro‐ and nanostructured systems that exhibit enhanced therapeutic efficacy while minimizing side effects due to their inherent biocompatibility, ability to penetrate biological barriers efficiently, and active targeting abilities.^20^, ^21^ However, diverse sources of inspiration and design principles confer distinct characteristics upon these systems, thereby influencing their progress in laboratory research and clinical translation to some extent. Hence, it is imperative to comprehensively comprehend the attributes of various bioinspired micro‐ and nanostructured systems in cancer therapy and elucidate their underlying design inspirations and principles. This will significantly enhance our understanding of challenging scenarios encountered in the field. Notably, although some previous reviews have explored emerging applications of bioinspired micro‐ or/and nanostructured systems in biomedicine.^18^, ^22^ A comprehensive analysis on the classification and application of these systems specifically for cancer therapy is lacking, particularly regarding their potential for clinical translation.

## 2 CLASSIFICATION AND DESIGN PRINCIPLES OF BIOINSPIRED MICRO‐ AND NANOSTRUCTURED SYSTEMS FOR CANCER THERAPY

**FIGURE 2:**
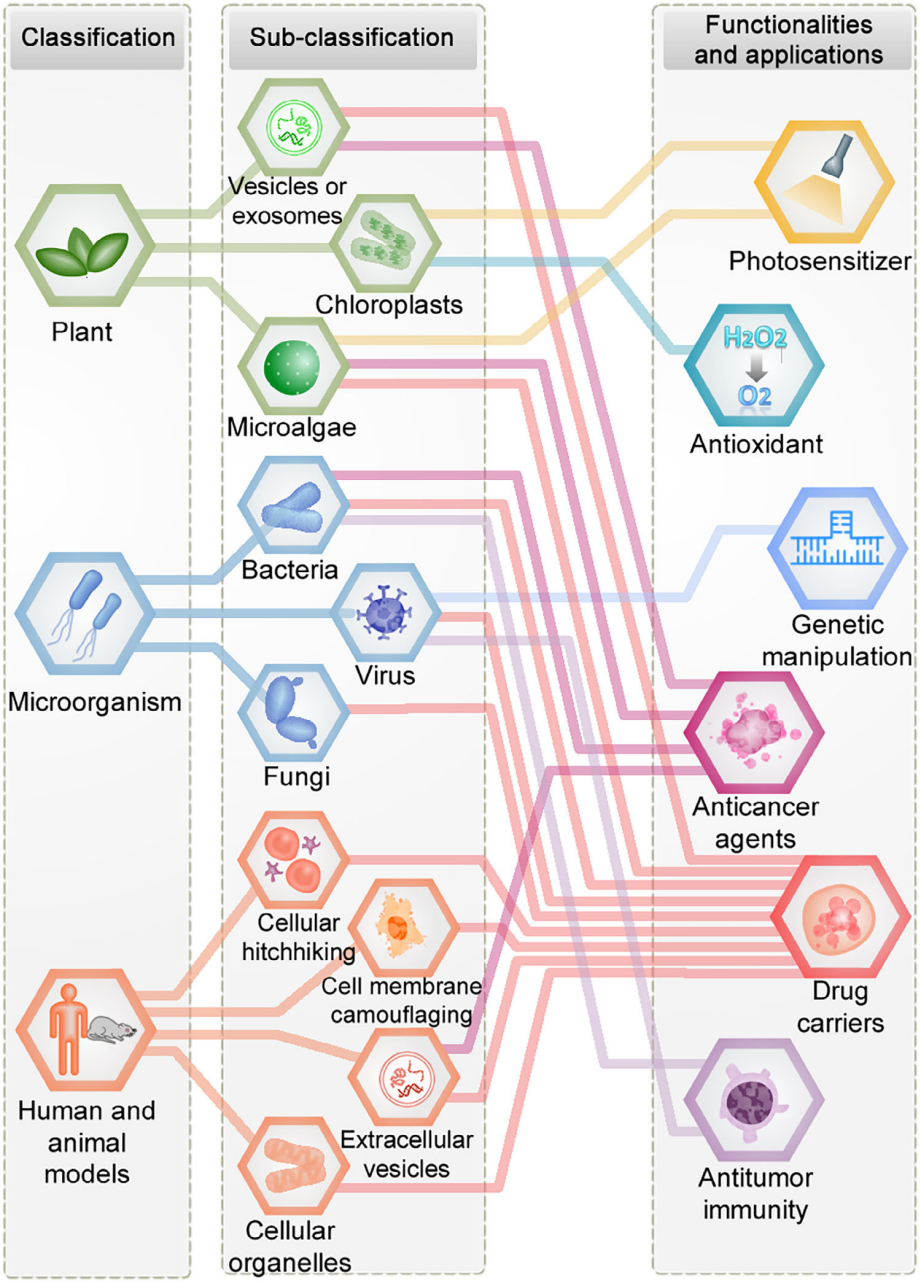
Global classification and applications of bioinspired micro‐ and nanostructured systems. The classification, subclassification, and applications of bioinspired micro‐ and nanostructured systems are presented with interconnected relationships.

Traditionally, biologists have classified organisms into the kingdoms of plants, microorganisms, and animals based primarily on morphological characteristics. In accordance with this conventional classification approach, our previous review introduced a novel categorization of bioinspired nanostructured systems as plant‐inspired, microbe‐inspired, and animal‐inspired drug delivery systems, with a specific focus on their applications in ocular diseases.^23^ In this section, we expand our classification approach to encompass the domain of cancer and present a pioneering contribution by proposing a systematic classification of bioinspired micro–nanostructured systems for cancer therapy (Figure 2). Furthermore, inspired by the natural world, our micro‐ and nanostructures are either directly or indirectly designed to emulate the biological functions and properties of plants, microorganisms, and animals, including structure, shape, locomotion, appearance, and surface characteristics. For the first time, we have combined principles of bioengineering, biocoupling, and micro–nanotechnology to introduce a subclassification system based on these design principles.

### 2.1 Plant‐inspired micro‐ and nanostructured systems for cancer therapy

Plants represent a fundamental life form, encompassing an estimated 350,000 extant species that include diverse organisms such as trees, shrubs, vines, grasses, ferns, green algae, and lichens. The direct inspiration from plant components has led to the development and application of various plant‐based therapeutics in cancer treatment, notably in chemotherapy, photodynamic therapy (PDT), immunotherapy, and gene therapy. Specifically, plant‐derived bioactive compounds,^24^ plant‐based vaccines,^25^ microRNAs,^26^ and extracellular vesicles (EVs)^27^ have been recognized for their therapeutic potential. However, since this paper focuses on the discussion of bioinspired micro‐ and nanostructured systems only, the exploration of vaccines, plant‐derived bioactive compounds, unmodified plant‐derived vesicles (PDVs), and microRNAs will be excluded from further analysis. Additionally, plants’ biological functions and characteristics like photosynthesis and shape have inspired the creation of diverse micro‐ and nanostructured systems tailored for cancer therapy.^28^, ^29^ This section will focus on the intricate design principles and biological functions of micro‐ and nanostructured systems inspired by the biological functions and performances of plant (including PDVs and photosynthesis). In addition to the inherent characteristics of bioinspired systems, such as relatively higher safety, targeting ability, and multifunctionality, the unique degradability, environmental friendliness, and cost effectiveness of plant‐derived systems endow them with potential in drug delivery and therapeutic efficacy.

#### 2.1.1 Plant‐derived vesicles

PDVs encompass a spectrum of lipid bilayer‐encapsulated vesicles and exosomes that are released by plant cells. These PDVs, including those derived from edible and medicinal plants, have emerged as promising agents in cancer therapy due to their low immunogenicity and biocompatibility.^27^, ^30^, ^31^ Unlike animal‐derived EVs, PDVs can be obtained in substantial quantities through advanced techniques such as ultracentrifugation, density gradient centrifugation, ultrafiltration, size‐exclusion chromatography, electrophoresis, polyethylene glycol (PEG) precipitation, and microfluidic engineering. This is possible due to the abundant availability of plant resources and the feasibility of in vitro cultivation. PDVs are characterized by their surface or encapsulated bioactive constituents such as small RNAs, proteins, lipids, and metabolites that facilitate internalization by recipient cells (particularly tumor cells) through various endocytic pathways.^32^, ^33^, ^34^

In the context of cancer therapy, PDVs have demonstrated utility both as drug carriers and direct anticancer agents. While Qiang et al. have provided a comprehensive overview of plant‐derived EVs in cancer therapy,^35^ our table distinctively encapsulates the latest applications and targeted delivery approaches of these PDVs, highlighting the specificity of biochemical content and administration methods tailored for various cancer type (Table 1). The ability of PDVs to traverse biological barriers (including the blood–brain barrier [BBB]) endows them with broad tumor‐targeting potential both in vitro and in vivo.^36^ Qiang et al.’s systematic evaluation and meta‐analysis demonstrate the therapeutic potential of PDVs in cancer therapy, highlighting their advantageous characteristics including low cytotoxicity and immunogenicity, high yield, capacity to modulate tumor cell behavior and the microenvironment, as well as oral administration being a safe and preferred delivery method.^35^ The investigation of PDVs in oncology underscores their versatility as drug delivery platforms and their contribution to the innovation of novel anticancer therapeutics. Further research is warranted to fully exploit their capabilities and translate these findings into clinical applications.

**TABLE 1:** PDVs for cancer therapy.

Source	Target cancer	Diameter (nm)	Biochemical content	Delivery method	References
Watermelon	Ovarian cancer	110–300	miRNA146	Intraperitoneal intraperitoneally	^32^
Broccoli	Human colorectal adenocarcinoma cells	35–300	miRNA	Cell culture	^37^
Apple, orange, pomegranate, and broccoli	Human colorectal adenocarcinoma cells	50–500	Exogenous miRNAs	Cell culture	^38^
Kiwi fruit	Non‐small cell lung tumor	∼198	siSTAT3	Intravenous injection	^39^
Celery (Apium graveolens l.)	Lung cancer	100–200	DOX	Intraperitoneal administration	^40^

Abbreviations: Cer, ceramides; DOX, doxorubicin.; PC, phosphatidylcholine; PDVs, plant‐derived vesicles; STAT3, signal transducer and activator of transcription 3; TG, triglycerides.

#### 2.1.2 Chloroplasts

In green plants, vegetable leaves are abundant in chloroplasts, which consist of thylakoids and stroma. The thylakoid membrane is rich in chlorophyll content. When exposed to environmental stresses such as high salinity and low temperature, chloroplasts accumulate H_2_O_2_.^41^ To prevent oxidative damage at this stage, the thylakoid membrane possesses catalytic activity to decompose toxic H_2_O_2_ into O_2_ through its association with catalase enzymes.^41^ Moreover, under light irradiation, chlorophyll can generate 1O_2_, making it a promising natural photosensitizer for PDT.^42^ Inspired by the unique structure and function of chloroplasts, Ouyang et al.^43^ successfully isolated complete components of the thylakoid membrane from chloroplasts and developed a biomimetic nanothylakoid capable of efficiently catalyzing the decomposition of endogenous H_2_O_2_ into O_2_ within tumor to overcome tumor hypoxia. Additionally, when exposed to near‐infrared light irradiation, the abundant chlorophyll within the thylakoids further converts O_2_ into highly toxic 1O_2_ to induce apoptosis in tumor cells. Drawing inspiration from the photonic biosystem of chloroplasts, Guo et al.^44^ developed a nanoplatform composed of Cu(II)–chlorophyll–hyaluronic acid (HA) nanoparticles (Cu(II)Ch–HA NPs), which demonstrates excellent synergistic application potential for cancer treatment through PDT and photothermal therapy (PTT), owing to its ability to generate singlet oxygen and exhibit remarkable photothermal conversion efficiency. Due to their high biosafety profile, low cost implications, and facile preparation methods, chloroplasts hold significant promise for clinical translation.

#### 2.1.3 Microalgae

Microalgae, as a rich source and lower member of green plant, have demonstrated significant therapeutic potential in cancer treatment by producing various compounds with anticancer properties, such as fucoidan and monoacylglycerides. These compounds exhibit inhibitory effects on angiogenesis and metastasis while selectively inducing apoptosis in cancer cells.^45^ In the field of drug delivery, micro‐ and nanoparticles inspired by microalgae's unique 3D structures have been utilized for targeted delivery of chemotherapeutic agents to enhance their efficacy and minimize side effects on normal cells. Encapsulation of anticancer drugs within microalgae‐inspired micro‐ and nanoparticles has emerged as a promising strategy to improve water solubility, prolong circulation half‐life, and facilitate better drug accumulation in tumor tissues.^46^ Recently conducted research suggests that Spirulina platensis, a microalga species utilized as a carrier for the radioprotective agent amifostine, has successfully developed an oral drug delivery system. This innovative system not only effectively shields the small intestine from radiation‐induced injury and prolongs survival without interfering with tumor regression but also promotes the balance of gut microbiota and exhibits long‐term safety, thus displaying promising prospects for potential clinical application.^47^

Similar to higher plants, microalgae possess the ability to undergo photosynthesis, utilizing light and chlorophyll to convert water and carbon dioxide into oxygen and glucose, which are crucial for growth. The rapid proliferation of tumor cells and alterations in metabolic pathways result in a persistent hypoxic state within solid tumors. This hypoxic microenvironment not only facilitates tumor invasion and metastasis but also contributes to resistance against chemotherapy, radiotherapy, and immunotherapy.^48^ Drawing inspiration from the photosynthetic process in microalgae, numerous research teams have commenced exploring the utilization of microalgae's photosynthetic capacity to ameliorate the hypoxic TME and enhance its sensitivity toward treatment.^49^, ^50^, ^51^ In a recent study conducted by Hua et al.^52^ a whole‐cell inorganic‐biohybrid system comprising Spirulina platensis and gold nanoclusters was developed. This system can enhance cancer radiotherapy through multiple pathways, including cascade photocatalysis. Initially, it generates oxygen under light irradiation followed by conversion of some oxygen molecules into superoxide anions (•O_2−_), subsequently oxidizing glutathione (GSH) within tumor cells. The combination of hypoxic regulation, •O_2−_ production, GSH oxidation along with radiotherapy sensitization induced by gold nanoclusters effectively enhances radiotherapy efficacy demonstrating superior antitumor effects in both 4T1 and A549 tumor models. Another study reported by Wang et al.^53^ involved the construction of micro‐oxygen factories known as photosynthesis microcapsules (PMCs), achieved through encapsulation of cyanobacteria and upconversion nanoparticles within alginate microcapsules. This system facilitates a sustained oxygen supply by converting external radiation into red‐wavelength emissions to support photosynthesis in cyanobacteria. Treatment with PMC suppresses the NF‐kB pathway, inhibits HIF‐1α production, and reduces cancer cell proliferation. The in vivo implantation of PMC creates a hyperoxic microenvironment that effectively hinders hepatocarcinoma growth and metastasis, exhibiting synergistic effects when combined with anti‐PD‐1 therapy in breast cancer. Overall, microalgae present a multifaceted approach to cancer therapy encompassing direct anticancer activity as well as the augmentation of drug delivery systems.

### 2.2 Microorganisms inspired micro‐ and nanostructured systems for cancer therapy

Microorganisms primarily consist of bacteria, viruses, fungi, and microalgae; the latter being categorized as plant‐inspired systems in the preceding section. In this section, our focus lies on the micro‐ and nanostructured systems inspired by bacteria, viruses, and fungi. The inspiration behind these systems is primarily derived from the inherent tropism of microbes toward tumor tissues, their capacity to navigate complex environments, modulation of immune responses, and in situ production of therapeutic agents.^54^ By employing sophisticated genetic and biochemical manipulations, the integration of microorganisms with advanced materials and technologies facilitates the development of bioinspired micro‐ and nanostructured systems that hold great potential for precise tumor tissue targeting, effective delivery of therapeutic agents, mitigation of side effects or systemic toxicity, as well as enhancement of therapeutic efficacy against diverse tumor type.^55^ Furthermore, compared with plant‐inspired systems, microorganisms naturally possess micrometer‐scale structures without the need for additional manipulation. Some microorganisms can actively home in on cancerous tissues and multiply within the tumor environment. They can be quickly cultivated and propagated in controlled settings, and genetic engineering operations are more readily performed to functionalize their surfaces, thereby achieving precise targeting capabilities. In this section, we present a comprehensive overview of micro‐ and nanostructured systems that are inspired by the characteristics and functionalities exhibited by bacteria, viruses, and fungi.

#### 2.2.1 Bacteria

*Living bacteria*: Typical examples of bacteria‐inspired micro‐ and nanostructured systems utilize bacteria themselves as therapeutic agents. Anaerobic or facultative anaerobic bacteria, as living organisms, can actively exit the vascular system using their bio‐motility and tend to migrate toward tumor tissue regions.^56^ The unique mechanisms by which live bacteria target solid tumors encompass two primary aspects.^57^, ^58^, ^59^ On the one hand, the sufficiently hypoxic, biochemically unique and immunoprivileged microenvironments resulting from pathological changes associated with solid tumors create favorable conditions for the growth of certain anaerobic bacteria; on the other hand, the intrinsic characteristics of bacteria, such as the preference of anaerobic bacteria to colonize tumor tissues rather than hypoxic or inflammatory lesions unrelated to tumors. Certain bacteria, like *Listeria*, possess distinctive tumor‐targeting mechanisms, enabling them to infect antigen‐presenting cells, including monocytes, macrophages, and dendritic cells, as well as myeloid‐derived suppressor cells. These cells can selectively deliver bacteria to the TME. Inspired by these characteristics, *Salmonella*, *Clostridium*, *Listeria*, *Lactobacillus*, *Bifidobacterium*, *Escherichia*, and so on have been widely applied for targeted cancer therapy.^60^, ^61^, ^62^, ^63^, ^64^, ^65^ Furthermore, certain bacterial species such as magnetotactic bacteria (MTB) exhibit remarkable sensitivity to magnetic fields. This unique trait can be strategically employed not only to guide these bacteria with enhanced precision to the tumor site but also to suppress tumor growth through magneto‐mechanical oscillations under a swinging magnetic field.^66^ However, being foreign entities within the host organism, the innate antigens on the surface of bacteria and the toxins and metabolites they release inevitably lead to the inactivation and rapid clearance of administered microbes by the immune system. This ultimately diminishes therapeutic efficacy and potentially induces side effects. Benefiting from integrated advancements in genetic engineering and materials science techniques allow for knocking out virulence genes without affecting bacterial viability while complementing functional material advantages.^67^ By employing strategies such as physical integration, chemical integration, biological integration, and encapsulation of multiple microorganisms, the protocol ensures the preservation of microbial activity, shields microbial immunogenicity, and significantly amplifies the microbes’ capacity for cancer therapy.^68^ Recently, based on chemical integration and encapsulation of microorganisms techniques, Cao et al.^69^ successfully prepared US FDA‐approved lipid membrane‐coated bacteria using interfacial self‐assembly and cell membrane‐coated bacteria through extrusion methods. Utilizing genetic engineering and materials science techniques, micro–nano biorobots, which are fabricated by genetically engineered live bacteria modified with multifunctional magnetic nanomaterials, have shown significant potential for tumor diagnosis and treatment under the influence of a magnetic field.^70^, ^71^, ^72^ The surface nanocoating technique enhances bacterial resilience against adverse conditions and demonstrates promising therapeutic efficacy and biosafety in mouse models following oral and intravenous administration, providing a versatile platform for advancing oncological treatments based on living bacteria, regardless of strain specificity.

**FIGURE 3:**
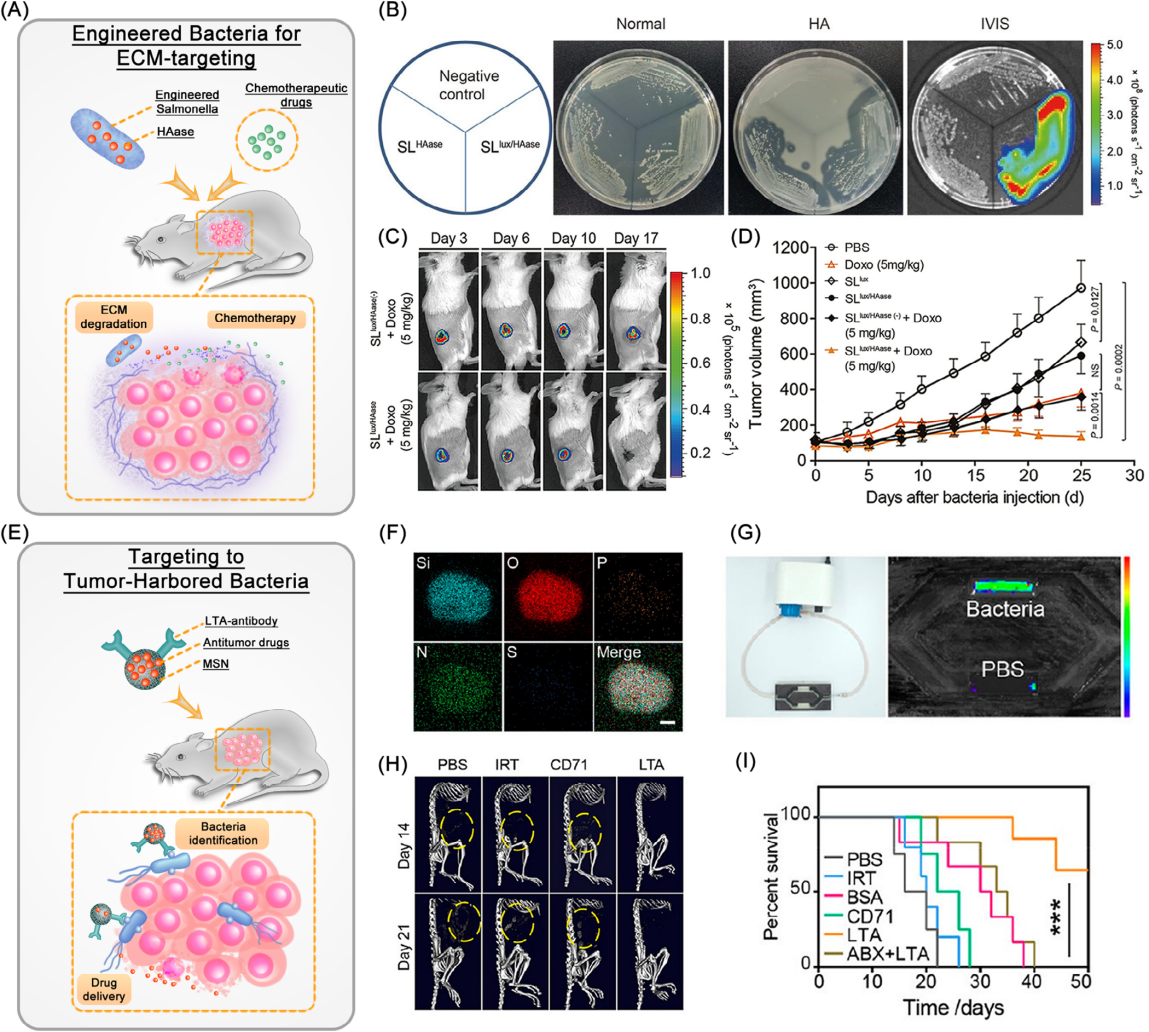
Bidirectional designs and applications of bacteria based cancer therapy. (A) Schematic illustration of the engineered bacteria based ECM‐targeting and cancer therapy. (B) Measurements of HAase activity in engineered HAase‐expressing bacteria. (C) Representative noninvasive in vivo imaging of bacterial bioluminescence. (D) Graphs depicting changes in the tumor volume over time after injections with bacteria. (E) Schematic illustration of the tumor‐harbored bacteria based targeting and cancer therapy. (F) HAADF–STEM images of LTA–MSNs. (G) Targeting ability of LTA–MSNs to bacteria in a flow environment. (H) CT scanning of tumor‐bearing mice at 14 and 21 days of the treatment. (I) Survival curves after receiving various treatments. Panels B–D were reproduced from Ref. 74 with permission from Elsevier (license number 5875820460360), Copyright 2023. Panels F–I were reprinted (adapted) with permission from Ref. 78. Copyright 2022 American Chemical Society. HAase, hyaluronidase; HA, hyaluronan; SLlux, Salmonella carrying an empty vector; SLHAas, plasmid pBAD–HAase was transformed into ΔppGpp S. typhimurium; SLlux/HAase, ΔppGpp S. typhimurium harboring the luciferase operon; Doxo, doxorubicin; LTA, lipoteichoic acid; MSN, mesoporous silica nanoparticle; IRT, irinotecan; BSA, bovine serum albumin; ABX, antibiotic cocktail.

In addition to their unique tumor‐targeting ability, certain bacteria such as *Salmonella* exhibit distinctive metabolic activities that facilitate the production of substantial quantities of H_2_S and acidic substances through anaerobic respiration. By utilizing this attribute, Wu et al.^73^ engineered a PEGylated Cu_2_O nanoparticle conjugated *Salmonella typhimurium strain*, termed Cu_2_O@ΔSt. This innovative approach harnesses the metabolic activity of bacteria to generate a substantial amount of H_2_S within the TME, facilitating the in situ conversion of Cu_2_O into copper with augmented photoacoustic imaging and photothermal properties. Consequently, this strategy significantly enhances the efficacy of PTT for localized tumors. Moreover, the genetic modification of *Salmonella* enables the bacteria to express specific enzymes that are adept at breaking down the ECM, which acts as a therapeutic barrier surrounding cancer cells. Specifically, through the genetic engineering of *Salmonella* to include the hyaluronidase (HAase) gene sequence, the bacteria are capable of secreting hyaluronidase proteins that efficiently degrade HA, a key component of the ECM. This targeted degradation significantly enhances the delivery of chemotherapeutic agents directly to the tumor site, thereby amplifying the therapeutic efficacy against cancer (Figure 3A–D).^74^ However, the implementation of multiple modification techniques may potentially escalate the treatment expenses. With the advent of gene sequencing technology, advancements in microbiome research methodologies, and the initiation of the Human Microbiome Project, it has been revealed that tumor tissue, previously believed to be sterile, harbors a distinct microbial community with varying abundance compared with normal tissue. This unexpected guest, which naturally thrives in the TME, is believed to exert a substantial impact on cancer therapy. Goto et al.^75^ successfully isolated and characterized three distinct bacterial strains from colon cancer tissues: *Proteus mirabilis* (A‐gyo), the photosynthetic bacterium *Rhodopseudomonas palustris* (UN‐gyo), and a composite bacterium comprising a synergy of A‐gyo and UN‐gyo. The inoculation of these bacteria into mice demonstrated high biocompatibility and potent antitumor capabilities. In addition, studies have shown that bacterial induced T cell immunity contributes to antitumor immune responses.^76^ Huang et al.^77^ developed tinidazole silver complex (antibiotics) loaded‐liposome for in situ delivery to bacterially infected mouse models of colorectal cancer (CRC). The targeted delivery of antibiotics within CRC tumors eradicates anaerobic bacteria and stimulates a CD8^+^ T cell‐mediated immune response against cancer cells. In addition to the antitumor approach that targets the elimination of tumor‐resident bacteria, inspired by the significant difference in bacterial counts between cancerous and normal tissues, Song et al.^78^ have developed bacteria‐targeting mesoporous silica nanoparticles modified with bacterial lipoteichoic acid (LTA) antibodies (LTA–MSNs). These nanoparticles can precisely target bacteria within tumors and deliver antitumor drugs for highly effective cancer therapy (Figure 3E–I). These naturally biocompatible tumor‐resident bacteria possess stronger innate therapeutic effects and higher tumor specificity, thereby effectively reducing treatment costs without the need for genetic manipulation or nanotechnology. In summary, as reviewed by Kwon et al.^79^ in addition to their inherent antitumor effects, local living bacterial therapies also induce innate and adaptive immune responses against cancer cells. This includes the activation of immune systems, promotion of immune cell infiltration, facilitation of the transition of the TME from “cold” to “hot,” and the release of cytokines and chemokines to attract and activate additional immune cells. Ultimately, these mechanisms lead to the death of tumor cells and the regression of tumors, either directly or indirectly. Such mechanisms offer novel perspectives and approaches for cancer therapy.

*Bacterial membranes and bacterial derivatives*: Another source of inspiration for bacterial‐based micro–nano systems is derived directly or indirectly from various components of bacteria, such as intact bacterial membranes (known as bacterial ghosts), as well as their derivatives including bacterial membrane vesicles (BEVs) and hybrid membrane biomaterials. Bacterial ghosts are generated through the process of gene E‐induced lysis and expulsion of cytoplasmic contents from Gram‐negative bacteria. This renders them noncytotoxic and safer alternatives to attenuated bacteria, making them effective delivery platforms for therapeutic agents such as vaccines and drugs. Among the bacterial derivatives, BEVs arise through outer membrane blebbing, known as membrane vesicles or cytoplasmic vesicles in Gram‐positive bacteria, while they are called outer membrane vesicles (OMVs) in Gram‐negative bacteria. Specially, OMVs have demonstrated distinctive characteristics such as membrane stability, immunogenicity, safety, penetration ability, and tumor targeting, which have been harnessed for the development of vaccines and drug delivery systems.^80^ Engineered OMVs have been shown to further reduce toxicity to normal cells, enhancing the safety and efficacy of treatments.^81^ Recently, wild‐type bacterium Escherichia coli Nissle 1917 (EcN) was genetically engineered by Chen et al.^82^ to overexpress tyrosinase, catalyzing the synthesis of melanin within cells, resulting in the production of melanin‐loaded OMVs. These were further coated with calcium phosphate, which not only reduced systemic inflammatory responses and damage to healthy organs such as the liver, spleen, lungs, and kidneys in mice but also, when combined with tumor photothermal/immunotherapy, significantly enhanced the overall antitumor effects. Hybrid membrane biomaterials can be created by mixing and compressing lysozymal‐degradated bacterial or OMV with other membranes. Delivery platforms based on these bacterial derivatives retain numerous advantages, including the ability to colonize and target tumors, enhance vaccine immunogenicity, and exhibit strong loading capabilities.^83^, ^84^ Recently, Chen et al.^85^ engineered a biomimetic nanovaccine by integrating toll‐like receptor 9 agonist CpG oligonucleotides, *Fusobacterium nucleatum* membranes, and liposomes prepared by 1,2‐dipalmitoyl‐sn‐glycero‐3‐phosphodayine, cholesterol and polyethylene glycol‐2000 conjugated 1,2‐distearoyl‐sn‐glycero‐3‐phosphoethanolamine using a using the classical thin‐film hydration process. This novel vaccine can selectively target and eliminate tumor cells while significantly augmenting chemotherapeutic outcomes for *Fusobacterium nucleatum*‐infected cancers and inhibiting cancer metastasis propensity. Furthermore, the hybrid membrane formulation can overcome the immunogenicity deficit inherent in bacterial vaccines that primarily consist of serotype‐specific polysaccharides and proteins, which typically elicit a subdued immune response. Additionally, researchers have also investigated the potential of other bacterial derivatives like bacterial spores,^86^ magnetosomes,^87^ and minicells^88^ as integral components in oncotherapy‐focused nanomedicine applications to harness distinct benefits offered by each component.

*Bacterial flagella*: Flagella play a pivotal role in eliciting the host immune response during bacterial infection. In *Salmonella typhi*‐mediated tumors, flagella can actively contribute to the host's immune response by promoting the expression of tumor necrosis factor‐alpha, a potent hemorrhagic agent.^89^, ^90^ Based on this pivotal function of flagella, Xu et al.^91^ ingeniously employed genetic engineering techniques to develop a novel tumor‐targeting bacterium called flhDC‐VNP. Upon colonization of tumors, flhDC‐VNP generates flagella that induce precise hemorrhaging and subsequent phagocytosis of red blood cells (RBCs) by tumor‐associated macrophages. This process releases hemoglobin that forms a complex with artemisinin, sustaining reactive oxygen species production and thereby exerting an anticancer effect. Moreover, the flagellar motor of bacteria serves as a nanoscale biological machine that can efficiently convert energy from ion gradients into mechanical motion, offering valuable insights for developing novel types of nanomachines. Martel et al.^92^ proposed a medical intervention approach utilizing flagellar MTB (e.g., MC‐1 MTB) to propel and guide computer‐controlled nanorobots toward deep‐seated tumors in the human body.

#### 2.2.2 Viruses

*Living viruses*: Viruses, as nanoscale pathogens with lipid envelopes derived from host cell membranes, exhibit high specificity for host cells and an invasive capability, highlighting their potential in targeted therapies. The protective protein or lipid shell ensures the integrity of their genetic material, which allows for easy genetic manipulation due to its compact size, thereby enhancing their utility in oncology for selective tissue targeting and therapeutic immune modulation.^93^ Tumor‐treating viruses are primarily classified into two groups: oncolytic viruses (OVs) and non‐OVs. OVs have emerged as a novel cancer therapeutic approach capable of selectively replicating within cancer cells and delivering various eukaryotic transgenes. This process induces immunogenic cell death and stimulates antitumor immunity. Currently, OVs including rigvir (ECHO‐7, discontinued owing to manufacturing issues in 2019), Talimogene laherparepvec (T‐VEC), and non‐OVs Nadofaragene firadenovec have been respectively approved for cancer treatment by US FDA in 2004, 2015, and 2022.^94^ However, the clinical use of OVs, which are often animal viruses, raises concerns due to the uncontrollable nature of viral replication and their pathogenicity.^95^ Genetic modification strategies for OVs involve the insertion of tumor‐specific promoters to enhance viral replication within cancer cells,^96^ the incorporation of tumor‐associated antigen targets to direct the virus toward cancer cell surface molecules,^97^ and the inclusion of microRNA response elements to restrict viral replication in normal cells.^98^ These modifications preserve the oncolytic and immunogenic properties of the virus while reducing its pathogenicity, offering a novel approach to decrease the toxicity associated with oncolytic virotherapy. Despite efforts to genetically modify them to reduce virulence, safety issues cannot be completely disregarded. Moreover, attenuated or inactivated viruses used as vaccines still pose safety concerns that may lead to severe allergic reactions or even disease recurrence or exacerbation.^99^ In contrast, plant‐derived viruses offer a safer alternative for applications in in vivo imaging and therapeutic delivery due to their lack of pathogenicity and infectivity in humans. Notably, recent research has demonstrated that the tobacco mosaic virus can induce M1 macrophage polarization, thereby effectively suppressing 4T1 breast cancer progression in murine models.^100^ Additionally, the brome mosaic virus has demonstrated low immunogenicity making it a potential vector for cancer therapeutics.^101^ Collectively these studies introduce novel paradigms and tools for the development of safer and efficacious cancer treatment methodologies.

*Virus‐like particles*: Virus‐like particles (VLPs), ranging typically from 20 to 200 nm in size, closely resemble the structure and appearance of viruses; however, they lack the genetic material necessary for viral replication. As highlighted by Chen et al.^102^ VLPs exploit the inherent structural antigens that make them indispensable in vaccine development and immunization strategies. Furthermore, VLPs have exhibited considerable potential in the field of drug delivery, emerging as promising carriers for a wide range of therapeutic agents, encompassing small molecule drugs, genetic therapies, peptides/proteins, and even nanoparticle‐based medications. Table 2 exemplifies the diverse applications of different VLPs in cancer therapeutics, elucidating their design principles and therapeutic approaches.

**TABLE 2:** Diverse applications of various VLPs in cancer therapeutics.

Viruses	Diameter	Design principle	Therapeutic method	Delivery method	Tumor	References
HBc VLPs	∼50 nm	The conjugate between the azido‐phenylalanine in HBc VLPs with the dibenzocycolctyne‐modified tumor‐associated antigens MUC1	Vaccine vectors	Subcutaneous	Lung metastatic mouse model	^103^
HBcAg‐33 VLP	–	Recombinant plasmid encoding a chimeric protein with mature IL‐33 molecules of HBcAg was inserted into the immunodominant epitope of expressed in E. coli DH5α cells and purified	Immunotherapy	Subcutaneous	4T1 breast cancer model	^104^
VLP–HA/VSP‐G and VLP‐HA	165 and 137 nm	Engineered VLPs pseudotyped with HA and NA from influenza A H5N1 or H1N1, with or without the coexpression of the extracellular region of VSPs fused to the transmembrane domain and cytoplasmic tail of VSV‐G (VLP–HA/VSP‐G and VLP‐HA, respectively)	Vaccination	Oral	4T1 breast cancer model	^105^
VLP–OVA_B_ and VLP–OVA_T_	87 and 79 nm	OVA_B_ peptide and OVA_T_ peptide of OVA were used here as model antigens and fused individually at the C terminus of the CP, which allowed display on the surface of phage P22 particles	Immunotherapy	Inguinally immunized	EG.7‐OVA lymphoma model	^106^
AU‐011	–	HPV VLPs modified by IRDye 700DX using HOSu reactive groups.	PDT and immunotherapy	Intravenous injection	TC‐1 lung cancer model	^107^
Smart HBV‐based nanodevices	∼50 nm	HBV VLPs decorated with GE11 peptide, GFP, and yCD through using the SpyCatcher/SpyTag system	Chemotherapy	Cell experiment	Inflammatory breast cancer cells	^108^
NoV VP1–MUC1 VLPs	∼50 nm	Bioengineered nanostructures based on the NoV VLPs was present multiple copies of tumour‐associated form of MUC1 epitope on their surface and produced in the LEXSY system in *L. tarentolae* cells	Immunotherapy	Subcutaneous	BALB/c male mice	^109^
DOX@RGD–VLPs	<40 nm	Three different HPV core protein derived VLPs as delivery vectors for DOX	Chemotherapy	Intravenous injection	CT26 colon cancer model	^110^
neoVLPs	Nanoscale	A collection of neoepitopes was expressed in HIV‐1 Gag VLPs through structural neoepitope prediction	Vaccine vectors	Subcutaneous	B16‐F10 melanoma mouse model	^111^

Abbreviations: VLPs, virus‐like particles; CP, coat protein; E. coli, Escherichia coli; GFP, green fluorescent protein; HA, hemagglutinin; HBc, hepatitis B core; HBV, hepatitis B virus; HIV, human immunodeficiency virus; HOSu, N‐hydroxysuccinimide; HPV, human papilloma virus; LEXSY, leishmania tarentolae; MUC1, mucin‐1; NA, neuraminidase; NoV, norovirus; OVA, ovalbumin; OVA_B_, B epitopes of OVA; OVA_T_, T epitopes of OVA; PDT, photodynamic therapy; RGD, arginine‐glycine‐aspartic acid; VSP, variant‐specific surface proteins.

*Virus‐mimicking nanoparticles*: Compared with VLPs and live viruses, virus‐mimicking nanoparticles (VMNs) incorporate a higher proportion of artificially synthesized components alongside a diminished amount of native viral constituents, ensuring the absence of genetic material and replication capabilities, thus elevating their safety profile. Through meticulous design and self‐assembly during manufacturing, these nanoparticles demonstrate a high degree of structural controllability. The dimensions, morphology, and surface attributes of VMNs can be customized to align with specific applications, thus improving their operational efficacy and versatility.^112^ Furthermore, by fine‐tuning their composition and shell materials, these particles can mimic various viral functions such as immune evasion, tissue specificity, cellular internalization, and membrane fusion.^113^ In light of the significant therapeutic potential of RNA therapy in glioma treatment and the challenges posed by biological barriers like cell membrane permeability and tissue specificity in clinical application, Zhu et al.^114^ drew inspiration from the targeting ability exhibited by Japanese encephalitis virus. They developed a strategy involving lipid nanoenvironments that regulate protein conformation to construct VMNs for targeted delivery of small interfering RNA (siRNA) to glioblastoma (GBM) cells. This approach achieved effective penetration through the BBB, tumor‐specific aggregation at GBM sites, siRNA release within tumors leading to substantial inhibition of tumor growth without adverse effects. These findings lay the foundation for tailored VMNs in precise RNA therapeutics for GBM.

#### 2.2.3 Fungi

Fungi display a diverse range of biological functions and have attracted attention due to their potential involvement in cancer. Genetically modifying the fungal cell wall enables the presentation of tumor‐associated antigens, effectively transforming them into powerful vehicles for cancer therapy.^115^ Furthermore, due to their simple cultivation processes, intact morphology, nonpathogenic nature, and ease of engineering, fungi serve as excellent model systems for modifiable material frameworks. For instance, the cell wall composition of yeast cells is a rigid electronegative structure containing functional groups or residues, which readily facilitates binding with particles.^54^, ^116^ Recently, Li et al.^117^ developed an intercooperative biohybrid platform by assembling positively charged S_2_O_3_^2–^ intercalated CoFe layered double hydroxides (LDHs) onto the negatively charged surface of *Saccharomyces cerevisiae* through electrostatic interactions. The degradation of LDHs within the TME induced the exposure of yeast surface antigens, leading to effective immune activation at the tumor site and demonstrating significant inhibitory effects on tumor ablation and recurrence.

### 2.3 Animals inspired micro‐ and nanostructured systems for cancer therapy

The remarkable capacity of animals, particularly humans, to adapt and evolve in response to intricate microenvironments serves as a valuable source of inspiration for the investigation of micro‐ and nanostructured materials. Compared with plant‐ and microorganism‐inspired micro‐ and nanostructured systems, human‐inspired micro‐ and nanostructured systems boast more advanced structures and functionalities, superior biocompatibility and bioactivity, and a greater potential for clinical translation. Within the field of cancer therapy, the human body itself provides a direct source for innovative strategies by encompassing various cell types and their microenvironments. Cellular hitchhiking (including microstructured blood cells, stem cells, and germ cells) of therapeutic agents enables the execution of targeted cancer therapy.^118^, ^119^, ^120^, ^121^, ^122^ Disguising therapeutic agents with cell membranes or EVs enhances their biocompatibility and targeting precision while circumventing clearance by the immune system.^123^, ^124^, ^125^ Furthermore, the TME, characterized by acidic pH, high GSH and H_2_O_2_ concentration, elevated lactate levels, and hypoxia, provides unique stimuli for the development of micro‐ and nanostructured drug delivery systems.^126^, ^127^ Inspired by the high expression of H_2_O_2_ in tumor sites, Yu et al.^128^ designed a novel type of nanomotor powered by a cascade enzyme reaction of catalase and glucose oxidase, which possesses self‐propelling motion characteristics. This nanomotor propels the composite nanoformulation to autonomously and controllable move toward the deep‐seated tumor, achieving effective treatment of the tumor. However, a recent comprehensive review by Zhang et al.^129^ has extensively covered TME‐inspired delivery systems. Therefore, this section will primarily focus on elucidating the process of utilizing human cells to fabricate innovative micro‐ and nanostructured systems for cancer therapy. Due to the assessment of immune efficacy in certain research studies, tumor models often necessitate the utilization of immunocompetent mice, potentially resulting in murine‐derived cells, cell membranes, or EVs. Nevertheless, this does not compromise the significance and necessity of our review, thereby duly elucidated.

#### 2.3.1 Cellular hitchhiking

**FIGURE 4:**
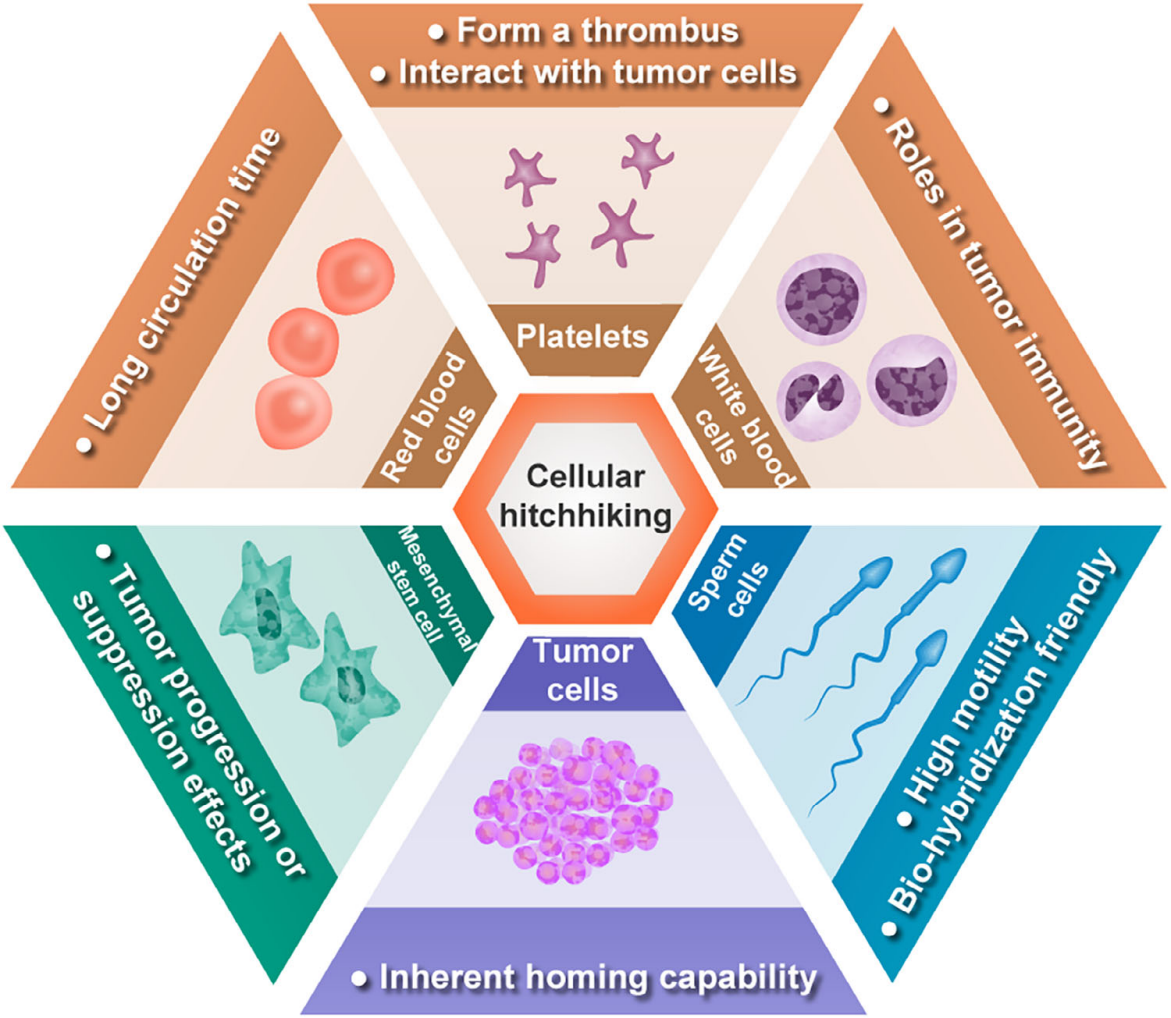
Overview of cell types and their distinctive features in the context of cell hitchhiking. The classification, cellular structures, and characteristics of various cell types are presented separately from the inner layer to the outer layer.

Cell hitchhiking, as defined by Zhou et al.^130^ refers to the process of disguising the cargo such as chemotherapeutics, mRNAs, vaccines and multifunctional nanoparticles as “self” to evade immune system clearance, overcome endothelial barriers, and extend circulation and reduce toxicity with minimized off‐target effects, thereby enhancing therapeutic efficacy against tumors. The concept of cell hitchhiking is based on the physiological characteristics of various cell types including blood cells, mesenchymal stem cells (MSCs), and tumor cells (Figure 4).

*Blood cells hitchhiking*: Blood cells encompass a variety of types, including RBCs responsible for oxygen transport, platelets involved in clot formation, and white blood cells (WBCs) responsible for immune response. RBCs possess notable drug delivery characteristics due to their long circulation time of up to 120 days, which has been successfully utilized as carriers for nanoparticles and bioactive agents such as enzymes, proteins, drugs, and macromolecules in cancer therapy. However, surface functionalization is often required to enhance the cargo's delivery capability due to the lack of active tumor targeting.^131^ Upon vascular injury, numerous platelets are recruited to the site to form a thrombus. Inspired by this phenomenon, engineered platelets have been utilized for targeted delivery of postoperative chemotherapy drugs and immune checkpoint inhibitors to mitigate tumor recurrence and postoperative metastatic dissemination.^132^, ^133^ Additionally, prior to tumor metastasis, platelets have exhibited interactions with tumor cells in the bloodstream, ascitic fluid, or microenvironment. Although these interactions offer potential insights for the treatment of metastatic tumors, they also entail certain risks due to the incomplete elucidation of underlying mechanisms.^134^ WBCs play an indispensable role in tumor immunity as immune cells that include lymphocytes, dendritic cells, monocytes/macrophages, granulocytes, and mast cells. Major approaches for hitchhiking immune cells can be categorized into four main methods: receptor modification, host cell genome engineering, therapeutic payload coengineering, and a combination of these methods.^135^ A prime example of receptor modification is the engineering of immune cells to express chimeric antigen receptor (CAR) capable of recognizing cancer cells, followed by their reinfusion into patient to exert antitumor effects. This breakthrough has led to significant advancements in adoptive cell therapy for immunotherapy, including CAR T cells (CAR‐T), CAR NK cells (CAR‐NK), and CAR macrophages (CAR‐M).^136^ Host cell genome engineering, exemplified by T cell receptor (TCR)‐T cell technology, is a method in which new genes are introduced into T cells to enable the expression of tumor‐specific TCRs, facilitating targeted recognition and elimination of cancer cells. This approach demonstrates an enhanced safety profile compared with CAR‐T therapy and represents an innovative strategy in the field of immunotherapy.^137^ Inspired by the ability of cancer cells to recruit inflammatory immune cells for their survival, Lee et al.^138^ have developed an in vivo therapeutic payload coengineering strategy using bioorthogonal click chemistry to modify CD11b antibodies on silica nanoparticles’ surface. This modification enables specific binding to CD11b^+^ myeloid cells, allowing efficient delivery deep into tumor regions and overcoming the challenge of limited penetration faced by nanocarriers. Adenosine production within the suppressive TME poses a limitation for CAR‐T therapy efficacy against solid tumors. In an excellent study conducted by Siriwon et al.,^139^ a combination approach involving receptor modification and therapeutic payload coengineering was presented. Specifically, CAR‐T cells have been engineered to incorporate SCH‐58261 (a specific small molecule inhibitor of the adenosine receptor)‐loaded liposomal vesicles, creating CART.cMLV(SCH). These modified cells actively deliver therapeutic agents deep into the immunosuppressive TME and enhance CAR‐T therapy effectiveness against solid tumors. After 20 days of treatment for the SKOV3.CD19 tumor‐bearing mice, there was no statistically significant difference in tumor volume between the CAR‐T therapy group and the phosphate buffer‐treated control group (*p* > 0.05). However, the tumor volume in the control group was more than three times larger than that in the CART.cMLV(SCH) group, a difference that was statistically significant (*p* < 0.0001). Furthermore, compared with the CAR‐T therapy group, the CART.cMLV(SCH) treatment group exhibited a significantly extended median survival time (36 vs. 22 days, *p* < 0.0001). Consequently, this approach may potentially overcome a significant challenge encountered in the treatment of solid tumors with CAR‐T therapy.

*MSCs hitchhiking*: MSCs, predominantly derived from bone marrow, umbilical cord blood, and adipose tissue, are extensively utilized in both basic and clinical research on cancer treatment due to their tumor tropism and immune evasion capabilities.^140^ However, the role of MSCs in tumor growth is a matter of debate as they may have either tumor progression or suppression effects.^141^ In this section, with a focus on cell hitchhiking theme, we primarily emphasize the use of MSCs as a delivery system. Capitalizing on the inherent tumor‐tropic properties of certain MSCs, their genetic modifiability and susceptibility to various viral transductions have led to their engineering for targeted delivery purposes. These engineered MSCs are commonly employed for delivering suicide genes,^142^ therapeutic proteins (including interferon, proinflammatory cytokines, and apoptosis inducers),^143^, ^144^ drugs (chemotherapeutic agents and nanoparticles),^127^ as well as OVs (such as Newcastle disease virus, adenovirus and vaccinia virus),^145^, ^146^ for cancer treatment. Further investigation into the mechanisms underlying MSC behavior within the TME along with optimization strategies for utilizing them as effective delivery systems is crucial for advancing cancer treatment research.

*Tumor cells hitchhiking*: Tumor cells possess an inherent homing capability, yet the utilization of viable tumor cells as a cellular drug delivery system raises concerns due to potential oncogenic risks and safety issues. To mitigate these concerns, current studies have employed cryo‐shock treatment to inactivate tumor cells, subsequently harnessing them as vectors for antigen and adjuvant delivery in cancer immunotherapy.^147^ Notably, the new application of cryo‐shocked lung tumor cells as a CRISPR–Cas9 delivery system has demonstrated efficacy in synthetic lethality approaches targeting cyclin‐dependent kinase 4 gene editing in KRAS‐mutant non‐small cell lung cancer (NSCLC).^148^ Furthermore, the use of cryo‐shocked tumor cells loaded with oncolytic adenovirus, has shown promise in abrogating the proliferative and pathogenic capacities of tumor cells.^149^ Additionally, cryo‐shocked cancer cell microgels have been explored for posttumor surgery combination immunotherapy and tissue regeneration, offering a novel avenue for therapeutic intervention.^150^

*Sperm cells hitchhiking*: Sperm, traversing the physiological pathways of the reproductive system, have served as inspiration for the development of sperm‐inspired microrobots designed for targeted therapeutic delivery within female reproductive diseases.^151^, ^152^ In a groundbreaking study, the chemotherapeutic agent DOX was loaded onto sperm cells and equipped with a 3D‐printed magnetic tubular structure at the base of their tails. This innovative approach facilitated magnetic‐guided navigation of sperm to HeLa cell spheroids in vitro, enabling precise delivery of DOX.^153^ However, it is worth noting that the initial hitchhiking system using bovine sperm differs significantly in molecular composition from human sperm, which may limit its clinical applicability. Addressing this challenge, Xu et al.^121^ recently achieved successful loading of DOX into human sperm cells to create “human spermbots.” These microrobots were directed by magnetic microcapsules to deliver drug‐laden sperm specifically to cervical and ovarian cancer cells in a 3D culture model, demonstrating significant antitumor effects. These advancements underscore the promising potential of utilizing sperm‐based microrobots for targeted treatment of gynecological malignancies.

In conclusion, the concept of cell hitchhiking emerges as a burgeoning field in oncology, harnessing the intrinsic characteristics of cells to revolutionize drug delivery mechanisms, overcome biological barriers, and enhance the efficacy of therapeutic interventions while concurrently mitigating their toxic side effects. Recently, synthetic cells‐artificial constructs that encapsulate the molecular machinery necessary for transcription and translation—have been employed to synthesize anticancer proteins within tumors.^154^ Ongoing research is imperative for optimizing these cellular delivery systems and unraveling their complete therapeutic potential.

#### 2.3.2 Cell membrane camouflaging

Nanoparticles cloaked in cell membranes derived from various sources, including RBCs, WBCs, platelets, MSCs, cancer cells, or hybrid membranes, present a novel biomimetic platform that mimics the inherent or engineered biological functions of cellular membranes within biological systems.^155^ These nanoparticles camouflaged with cell membranes possess the cellular membrane's composition, including specific receptors, antigens, and proteins crucial for precise tumor targeting, immune evasion, and extended circulation time within the bloodstream.^156^ The engineering of these nanoparticles enables the selective capture of specific proteins, antigens, and receptors, thereby augmenting their functional capabilities. Recently, Yang et al.^157^ designed genetically programmable human embryonic kidney 293 T cells to express variants of signal regulatory protein alpha, which significantly enhances affinity between cell membrane‐camouflaged nanoparticles and CD47 on surface of colon cancer cells while retaining extended circulation characteristics of original cell membrane. This dual functionality allows utilization of these nanoparticles for targeted combination therapy in treatment of CRC. Furthermore, MPB‐3BP@CM nanoparticles designed to improve immunosuppressive TME when disguised with CAR T cell membranes act as catalysts to amplify the efficacy of CAR T cell therapy against solid tumors.^158^ In the realm of tumor targeting where homologous targeting capabilities are crucial, the use of tumor cell membranes circumvents the unpredictable potential of live cells to promote tumor development. For instance, in the case of osteosarcoma, which poses a targeting challenge, the utilization of K7M2 cancer cell membrane‐derived strategies has demonstrated remarkable efficacy.^159^ Hybrid membranes offer multifunctionality by combining different cell types; for example, the fusion of 4T1 tumor cells with RAW264.7 macrophages generates a cytomembrane that not only achieves efficient homologous tumor targeting but also facilitates effective antigen presentation and activation of effector T cells.^160^ Looking ahead, the ongoing advancement in genetic programming and hybrid membrane technology holds tremendous potential for overcoming current limitations in cancer treatment through cell membrane camouflaged nanoparticles. This progress may lead to more personalized and potent immunotherapies capable of precisely targeting and eradicating tumors while minimizing side effects.

#### 2.3.3 Extracellular vesicles

EVs, encompassing a diverse array of membrane‐bound vesicles released by cells, are gaining recognition for their potential in therapeutic applications and drug delivery systems.^161^ These “small but strong” vesicles encapsulate the same genetic material, proteins, and lipids as their parent cells, thereby preserving the inherent attributes of low immunogenicity, excellent biocompatibility, natural BBB penetration ability, and efficient gene delivery capability.^162^ They have exhibited both safety and efficacy in preclinical models as well as early clinical trials, positioning them as a promising next‐generation therapeutic modality and drug delivery platform.^163^ EVs can be directly isolated from cells through physical or chemical methods and engineered to possess tumor‐targeting therapeutic functions. Alternatively, they can be derived from genetically or metabolically engineered cells to produce directly engineered EVs.^164^

The complexity of EVs lies in their subpopulations, which can be categorized into four subgroups based on their diameter and mode of formation: microvesicles, larger EVs with diameters exceeding 150 nm formed through direct budding from the cell membrane; exosomes, smaller EVs measuring less than 150 nm; apoptotic bodies, a more intricate subset of EVs characterized by higher ambiguity in characterization and a size range of 50–5000 nm; and oncosomes, atypical large cancer‐derived EVs with diameters ranging from 1 to 10 µm that originate from membrane blebbing.^165^ Apoptotic bodies, generated during the final stages of apoptosis, have gained increasing research attention due to their potential in drug loading and therapeutic applications.^166^ As carriers, apoptotic bodies offer several advantages including high production efficiency and the ability to transport residual drugs to neighboring tumor cells after cellular apoptosis. This provides novel insights into drug loading methods and their applications in cancer therapy.^167^ Notably, nanoscale apoptotic bodies derived from the highly brain‐metastatic cell line B16F10 (murine melanoma cells) can traverse the BBB and are ultimately internalized by microglia in the brain. This offers a promising approach for targeted delivery of antitumor nanodrugs across the BBB to treat brain tumors.^168^ Inspired by monocytes/macrophages’ phagocytic action mediated by phosphatidylserine highly expressed on apoptotic body surfaces, Liu et al.^169^ engineered multifunctional apoptotic bodies that “hitchhike” monocytes/macrophages across the BBB. These engineered bodies effectively deliver gold nanorods and immune adjuvants loaded onto them to the tumor region, combining photothermal and immunotherapies for efficient elimination of tumor cells. Oncosomes have been shown to promote tumor growth and metastasis.^170^, ^171^ However, their tumor‐homing properties also provide a basis for targeted cancer therapy. Although oncosome research is still largely unexplored at present, with further elucidation of their mechanisms and functionalization through genetic engineering, they possess significant potential in the field of targeted cancer therapy. The potential of EVs as drug delivery systems is currently being explored, aiming to enhance their therapeutic efficacy and clinical applicability. As our understanding of EVs continues to expand, the transformative potential of these vesicles in the fields of therapeutics and drug delivery for oncology becomes increasingly apparent.

#### 2.3.4 Cellular organelles

Cellular organelles, fundamental structures of the cell, play a crucial role in maintaining normal physiological activities. Bioinspired organelle‐mimicking drug delivery systems, owing to their exceptional biocompatibility and superior drug‐loading efficiency, have found extensive applications across diverse domains encompassing anticancer therapy, treatment of neurodegenerative diseases, management of antimicrobial infections, wound healing, as well as organ disease therapeutics.^125^ Mitochondria, being the cellular powerhouses, possess inherent biocompatibility due to their endogenous origin. Taking inspiration from this, Li et al.^172^ isolated mitochondria from the breast cancer cell line MDA‐MB‐231 and successfully achieved targeted delivery of carbon quantum dots and the chemotherapeutic drug DOX to tumor sites. Recently, Zou et al.^173^ innovatively utilized a hybrid membrane fabricated from cancer cell and mitochondrial membranes to create a bioinspired nanosystem loaded with drugs, achieving a dual‐targeting effect where the hybrid nanoparticles are specifically absorbed by target cells and then target subcellular organelles. Additionally, Xu et al.^174^ successfully mimicked mitochondrial oxidative phosphorylation scenario using Au nanoparticle‐trapped hollow silica microspheres, demonstrating high oxidative phosphorylation activity comparable to that of natural mitochondria. In tumor‐infiltrating T cells, suppression of mitochondrial metabolism leads to diminished antitumor efficacy.^175^ Biomaterials that mimic mitochondrial oxidative phosphorylation could potentially facilitate the metabolic reprogramming of T cells, thereby enhancing their antitumor capabilities. Lipid droplets are nano‐ and microscale organelles in adipocytes that can be efficiently isolated while retaining critical physiological functions for interactions with other organelles. Inspired by this concept, Liang et al.^176^ loaded photosensitizers into lipid droplets for PDT. The accumulation of these lipid droplets induced oxidative stress and disrupted intracellular metabolic balance, ultimately triggering the apoptotic signaling pathway through endoplasmic reticulum stress and resulting in significant antitumor effects. However, the efficient delivery of therapeutic agents to the cell nucleus remains a major challenge due to strict regulation by nuclear pore complexes (NPCs) and importins. To address this issue, Nagahama et al.^177^ developed bioinspired nuclear nanotransporters using self‐assembled amphiphilic polysaccharide‐amino acid derivative conjugates that mimic the basic structure and chemical characteristics of importins in NPCs. These nanotransporters successfully delivered enzymes and synthetic anticancer drugs into the cell nucleus while preserving their bioactivity. In summary, biomimetic micro‐ and nanosystems inspired by the unique properties of organelles, including mitochondria, lipid droplets, and the cell nucleus hold great potential in precision oncology by enabling targeted drug delivery with maintained bioactivity through innovative strategies.

## 3 CLINICAL TRANSLATION AND CHALLENGES OF THE BIOINSPIRED MICRO‐ AND NANOSTRUCTURED SYSTEMS

**FIGURE 5:**
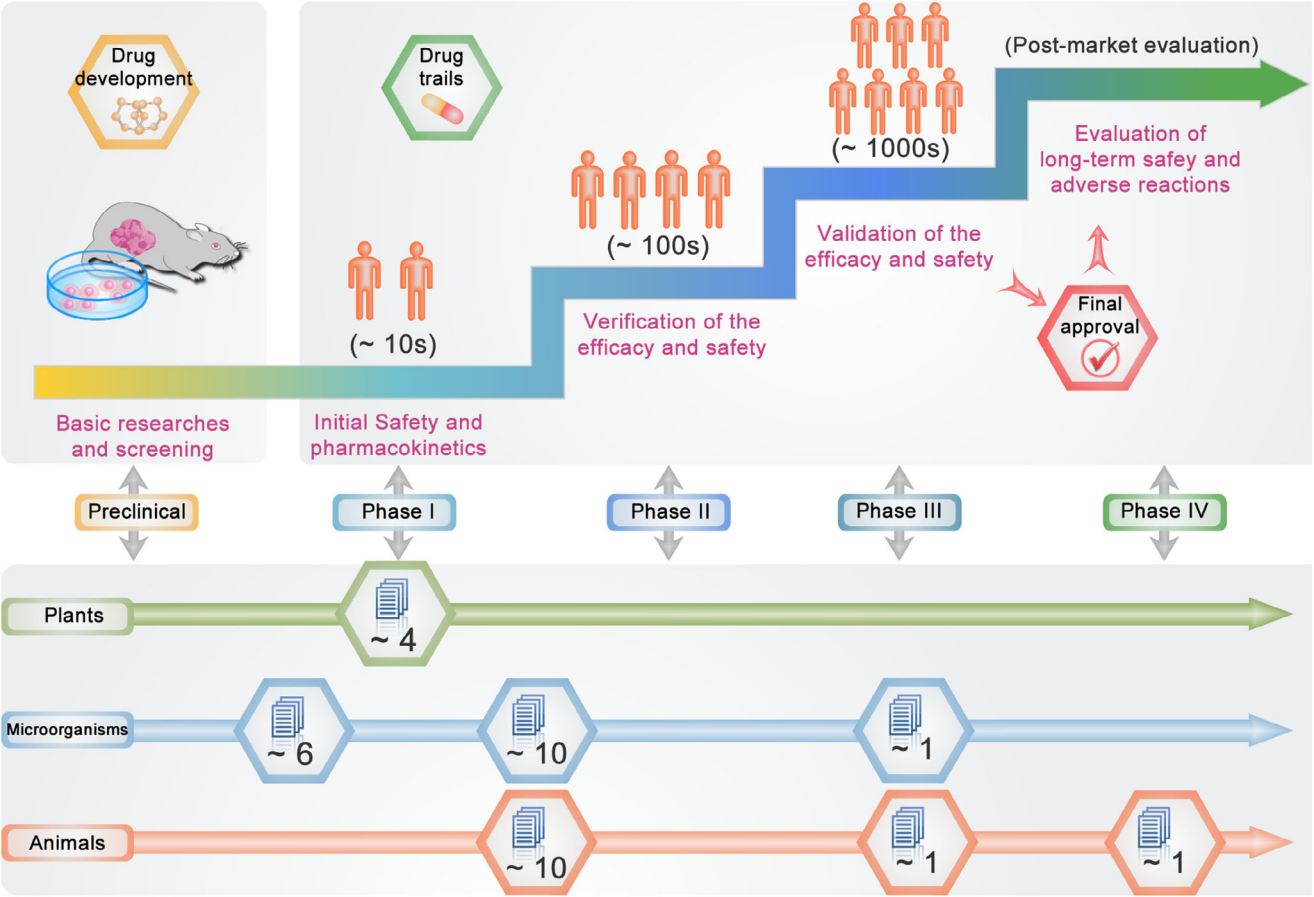
Overview of the stages and current status of clinical translation. The upper part of the figure depicts the general processes involved in developing clinical therapies, ranging from basic research to various stages of clinical trials. Meanwhile, the lower part illustrates the latest status of recently registered clinical trials for bioinspired micro‐ and nanostructured systems applications.

Considering the promising anticancer outcomes demonstrated in both in vivo and in vitro studies, the utilization of bioinspired micro‐ and nanostructured systems has emerged as a highly encouraging therapeutic strategy for cancer treatment. Several ongoing or completed clinical trials have yielded promising results, leading to the integration of some systems into clinical applications (Figure 5). However, certain challenges still persist. Subsequent sections will provide an in‐depth discussion on the current status of clinical transformation of micro‐ and nanostructured systems inspired by plants, microorganisms, and animals, as well as the encountered challenges during this process for the first time.

### 3.1 Clinical translation of plant‐inspired micro‐ and nanostructured systems

In the preceding chapters, we have introduced plant‐inspired micro‐ and nanostructured systems, encompassing PDVs, chloroplasts, and microalgae. To date, only four clinical trials related to PDVs have progressed to Phase I according to the www.clinicaltrials.gov website. Among them, one completed clinical trial investigating the potential of ginger‐derived vesicles (NCT04879810) in the elimination of inflammatory bowel disease and one withdrawn trial exploring the alleviation of ginger and aloe‐derived vesicles (NCT03493984) on insulin resistance and chronic inflammation in patients with polycystic ovary syndrome are not pertinent to cancer treatment, thus we will refrain from discussing them here. However, there was one ongoing Phase I clinical study (NCT01294072) that employed fruit‐derived vesicles for delivering hydrophobic anti‐CRC drug curcumin via oral administration to both normal and CRC tissues; unfortunately, this trial did not complete subject recruitment. Additionally, another trial (NCT01668849) aimed to evaluate the efficacy of grape‐derived vesicles in reducing oral mucositis incidence during radiotherapy and chemotherapy for head and neck tumors when administered orally. The first phase of this clinical trial has been completed but results are yet to be disclosed. No clinical trials investigating the potential of chloroplasts or microalgae for cancer treatment have been identified. Consequently, despite promising advancements in basic research regarding plant‐inspired micro‐ and nanostructured systems, their translation into clinical applications remains unrealized.

### 3.2 Clinical translation of microorganisms‐inspired micro‐ and nanostructured systems

#### 3.2.1 Bacteria‐inspired systems

The concept of utilizing bacteria‐inspired micro‐ and nanostructured systems for cancer therapy can be traced back to the 19th century. Recently, Gao et al.^178^ conducted a comprehensive survey on clinical applications of engineered bacteria or bacterial components in micro‐ and nanostructured systems for Phase I/II clinical cancer therapy over the past decade. Herein, we supplement this with an overview of micro‐ and nanostructured systems based on bacteria (mainly Bacillus Calmette‐Guérin, BCG) or bacterial components that have been widely applied clinically. These micro‐ and nanodrugs draw inspiration from live bacterial strains, attenuated bacteria, genetically modified bacteria, bacterial spores, and OMVs. They are employed in the treatment of various malignancies such as metastatic pancreatic cancer, malignant pleural mesothelioma, bladder cancer, and recurrent GBM multiforme. The therapeutic approaches involve administering bacterial preparations via intravesical, subcutaneous, intravenous, or oral routes; combining bacteria with therapeutic drugs; or designing strategies to elicit an immune response against tumor antigens.

We will now focus on the status and challenges of BCG, the first bacterial product successfully applied in cancer therapy. BCG is a live bacterial preparation derived from attenuated bovine tuberculosis bacilli suspension. It received US FDA approval in 1990 for treating bladder cancer. Although patients with nonmuscle‐invasive bladder cancer (NMIBC) responsive to this treatment exhibit high survival rates, approximately 35% of high‐risk NMIBC patients experience recurrence and are classified as BCG‐unresponsive NMIBC.^179^, ^180^ Radical cystectomy (RC) with pelvic lymph node dissection remains the standard treatment option; however, it is associated with significant perioperative morbidity and may not be suitable for many patients who relapse after BCG therapy. To address this dilemma, Hahn et al.^179^ conducted a Phase I clinical trial investigating the combination of Durvalumab and BCG for treating BCG‐unresponsive NMIBC patients. Among 13 subjects, the complete response (CR) rate at 3 months reached an impressive 85%, surpassing that of pembrolizumab (41%)^181^ and nadofarogene firadenovec (53%),^182^ both approved by US FDA for treating BCG‐unresponsive NMIBC patients. Furthermore, the CR rate at 12 months was remarkable at 73%. However, another Phase II clinical trial targeting the same disease reported a mere 12% complete response rate at 6 months.^183^ The inconsistency between these two studies could be attributed to variations in treatment methods and individual patient characteristics. Currently, other combination therapies for BCG‐unresponsive NMIBC patients are actively being investigated through clinical trials, such as the combination with the chemotherapy drug gemcitabine (NCT04179162). A comprehensive investigation into the mechanism of BCG therapy is crucial for advancing novel treatment modalities for refractory NMIBC. Martinez‐Lopez et al.^184^ recently conducted an in‐depth analysis of the mechanism of BCG vaccine therapy for bladder cancer and revealed that it potentially involves early recruitment and polarization of macrophages toward a proinflammatory phenotype. Consequently, the development of therapeutics targeting the innate immune system, particularly macrophages, holds great promise as a future avenue for addressing BCG‐unresponsive NMIBC.

The global shortage of BCG has prompted researchers to actively develop alternative strategies, drawing inspiration from other bacteria, for the treatment of NMIBC. In an ongoing multicenter, single‐arm Phase II clinical study (NCT05975151), the efficacy and safety of intravesical instillation of *Pseudomonas aeruginosa* following transurethral resection of bladder tumors are being evaluated in patients with intermediate and high‐risk NMIBC. However, patient recruitment for this trial is still underway. VAX014 is a recombinant *Escherichia coli* minicell that can rapidly permeate the cytoplasmic membrane of target cells while inducing apoptosis, and has been used for the treatment of NMIBC in mice.^185^ An ongoing study investigating the intravesical instillation of VAX014 in NMIBC has recently been completed; however, the results are yet to be disclosed (NCT03854721). Clinical investigations involving other bacterial derivatives such as bacterial ghosts and OMVs have not yet been reported.

#### 3.2.2 Virus‐inspired systems

The early treatment of viruses was associated with significant side effects and low response rates. Benefiting from the advancement of genetic recombination technology, four OVs and one non‐OV have been globally utilized for cancer therapy.^94^ For instance, Onyx‐015, the first engineered adenovirus to be approved for clinical application in the treatment of human head and neck carcinoma.^186^ T‐VEC, an engineered oncolytic herpes simplex virus type 1 (HSV‐1), has been designed to selectively replicate in tumor cells and induce antitumor immune responses. It is currently the only widely approved therapeutic method.^94^, ^187^ Furthermore, a Phase II clinical study led by Li et al.^188^ evaluated the efficacy of a serotype‐5 oncolytic adenovirus regimen incorporating CG0070 and pembrolizumab as a combined therapeutic approach for patients with BCG‐unresponsive NMIBC. This investigation demonstrated a commendable CR rate of 82.9% within 3 months, which closely approximates the 85% remission rate associated with BCG in conjunction with Durvalumab as detailed in Section 3.2.1.^179^ These encouraging outcomes not only provide a promising alternative for individuals who are refractory to standard BCG therapy but also have the potential to alleviate strain on existing BCG supplies. Table 3 presents more than ten novel virus‐based drugs that have emerged in clinical cancer therapy trials over the past year. It is worth noting that the HSV has also been the most frequently utilized virus in clinical trials from 2023 to August of 2024, with mature modification techniques for this virus. Despite the advancements, the risk of off‐target effects and unintended toxicity from genetic manipulation may persists, leading to stringent clinical trial criteria that exclude patients with impaired immune systems or active viral infections to mitigate safety concerns. Among viral components, VLPs have emerged as a prominent choice in clinical trials due to their superior safety profile compared with live viruses and other advantages. A recently published study investigating the use of VLP‐mediated delivery of anticancer drugs for PDT in choroidal melanoma demonstrated a remarkable 100% tumor control rate after 9 months, accompanied by an 88% preservation rate of visual acuity. Furthermore, the overall tolerability was excellent, with no instances of dose‐limiting toxicity, treatment‐related severe adverse events, or significant adverse events observed. These findings hold great promise for targeted vision‐protecting therapy as a first‐line approach in primary choroidal melanoma treatment (NCT04417530).

**TABLE 3:** Living virus‐inspired drug delivery platforms in clinical trials for cancer therapy from 2023 to August of 2024.

Inspiration	Name of the drug	Delivery method	Type of cancer	Study status	NCT number	Phases
HSV‐1	SDJ001 or YD06‐1	Intratumoral injection	Late stage solid tumors including squamous cell carcinoma of the head and neck, and malignant melanoma	Recruiting	NCT06080984	Phase I
HSV‐1	R130	Intratumoral or intraperitoneal Injection	Advanced solid tumors	Recruiting	NCT05860374	Early Phase I
HSV‐1	R130	Intratumoral or intraperitoneal	Advanced solid tumors	Recruiting	NCT05961111	Early Phase I
HSV‐1	R130	Intratumoral or intraperitoneal	Advanced solid tumors	Recruiting	NCT05886075	Early Phase I
HSV‐1	R130	Intratumoral or intraperitoneal	Advanced bone and soft tissue tumors	Recruiting	NCT06171282	Early Phase I
HSV‐1	R130	Intratumoral or intraperitoneal	Relapsed/refractory cervical and endometrial cancer	Recruiting	NCT05812677	Early Phase I
HSV‐1	R130	Intratumoral or intraperitoneal	Relapsed/refractory head and neck cancer	Recruiting	NCT05830240	Early Phase I
HSV‐1	R130	Intratumoral or intraperitoneal	Relapsed/refractory bone and soft tissue tumors	Recruiting	NCT05851456	Early Phase I
HSV‐1	STI‐1386	Intratumoral or intraperitoneal	Relapsed or refractory solid tumors including locally advanced pancreatic cancer, unresectable soft tissue sarcomas, hepatic metastases due to CRC	Not yet recruiting	NCT05361954	Phase I
HSV‐1	RP2/RP3 in combination with Atezolizumab and Bevacizumab	Intratumoral injection	CRC	Active not recruiting	NCT05733611	Phase II
HSV‐2	OH2	Transcatheter intraarterial infusion	Advanced liver cancer	Recruiting	NCT05698459	Phase I
HSV‐2	OH2	Intratumoral injection	Locally advanced/metastatic solid tumors	Not yet recruiting	NCT05954091	Phase I
HSV‐2	OH2	Intratumoral injection	Advanced colorectal cancer	Terminated	NCT05648006	Phase II
HSV‐2	OH2	Intratumoral injection	Melanoma	Recruiting	NCT05868707	Phase III
HSV‐2	oHSV2‐PD‐L1/CD3‐BsAb	Intramural injection	Advanced/metastatic solid tumors	Recruiting	NCT05938296	Phase I
HSV‐2	H101 combined with PD‐1	Intratumoral injection	Advanced malignant pleural mesothelioma	Recruiting	NCT06031636	Observational
Adenovirus	TILT‐123 and Avelumab	Intratumoral injection	Solid tumors refractory to or progressing after anti‐PD 1	Recruiting	NCT05222932	Phase I
Human adenovirus serotype 5	Ad‐TD‐nsIL12	Intratumoral injection	Primary pediatric diffuse intrinsic pontine glioma	Recruiting	NCT05717712	Phase I
Human adenovirus serotype 5	Ad‐TD‐nsIL12	Intratumoral injection	Progressive pediatric diffuse intrinsic pontine glioma	Recruiting	NCT05717699	Phase I
Wild‐type influenza strain A	CodaLytic	Intratumoral injection	Metastatic or otherwise inoperable breast cancer	Withdrawn	NCT05600582	Phase I
Vaccinia virus	KM1	Intraperitoneal infusion	Recurrent or refractory ovarian cancer	Recruiting	NCT05684731	Phase I
Vaccinia virus	hV01	Intratumoral injection	Advanced solid tumors	Recruiting	NCT05914376	Phase I
Vaccinia Virus	GC001	Intratumoral injection	Advanced solid tumors	Recruiting	NCT06508307	Phase I
Vaccinia virus	onCARlytics (CF33‐CD19) in combination with blinatumomab	Intravenously or intratumorally	Adults with advanced or metastatic solid tumors	Recruiting	NCT06063317	Phase I

*Data source*: ClinicalTrials.gov.

Abbreviations: CD, cluster of differentiation; CD3‐BsAb, CD3‐bispecific antibodies; CRC, colorectal cancer; HSV, herpes simplex virus; oHSV2, oncolytic herpes simplex virus type 2; PD‐1, programmed death receptor 1.; PD‐L1, programmed death ligand 1.

#### 3.2.3 Fungi‐inspired systems

Despite the potential of micro‐ and nanostructured systems inspired by fungi in cancer therapy, there is currently a lack of ongoing clinical trials investigating their application. Despite the potential of micro‐ and nanostructured systems inspired by fungi in cancer therapy, there is currently a lack of ongoing clinical trials investigating their application. However, the discovery of fungi within tumors and an improved understanding of how these intratumoral fungi interact with tumorigenesis and tumor progression offer a promising avenue for future research.^189^, ^190^, ^191^ By genetically modifying these fungi to eliminate key pathogenic genes and developing micro‐ and nanostructured systems based on their biology, targeted cancer therapies could be devised, providing a novel and potentially effective approach to treatment.

### 3.3 Clinical translation of animals‐inspired micro‐ and nanostructured systems

The clinical translation of cells hitchhiking system for cancer therapy has yielded mixed results. For instance, Eryaspase, the first engineered RBC encapsulating l‐asparaginase developed for the treatment of cancers, failed to meet its primary endpoint of improving overall survival in a Phase III clinical trial as a second‐line treatment for metastatic pancreatic cancer (NCT03665441). However, Eryaspase has received US FDA fast track designation for the treatment of acute lymphoblastic leukemia (ALL) due to positive results in a trial involving pediatric ALL patients allergic to PEG‐asparaginase, highlighting its potential application in this specific patient group (NCT03267030). A Phase I clinical trial, initiated in July of this year, aims to assess the safety, pharmacokinetics, pharmacodynamics, and efficacy of commercially available αPD‐1 conjugated with RBC in patients with advanced malignancies. Although patient recruitment has not yet begun (NCT06528249), this clinical trial represents a significant advancement in exploring the potential of erythrocyte‐conjugated αPD‐1 for cancer treatment. Clinical trials involving platelet hitchhiking have not yet initiated. In contrast to the limited clinical progress observed with the aforementioned cell types, immune cell‐based therapies are rapidly advancing toward clinical translation. According to recent statistical analysis by Han et al.,^192^ there has been a substantial increase in clinical studies focusing on CAR‐T, CAR‐NK, and TCR‐T since 2013; among them, CAR‐T cell therapy is the most matured field with 258 cases reported by 2022. CAR‐NK primarily targets hematological tumors while TCR‐T mainly investigates solid tumor treatment. Clinical research on CAR‐M is still at an early stage with three ongoing studies (NCT05007379, NCT04660929, and NCT06224738), including a 2024 study that explores Human HER2‐targeted CAR‐M for HER2‐positive advanced gastric cancer with peritoneal metastases. In recent years, MSC therapy trials for tumors have been relatively scarce and mostly involve natural MSC sources such as umbilical cord (NCT05672420) or adipose tissue (NCT05789394), as well as engineered MSCs used for tumor treatment research purposes. Among them, MSCs expressing therapeutic proteins TRAIL (NCT03298763) and INFα (NCT05699811) have recently been employed alongside other treatment modalities in Phase I and II clinical trials targeting NSCLC and locally advanced/metastatic solid tumors. However, due to ongoing participant recruitment, the results are currently unavailable. Other cell hitchhiking materials such as tumor cells and sperm cells have not been seen in clinical trials related to tumor treatment research.

Despite the significant advancements in cell mimetic platforms for clinical cancer therapy, there still remains a substantial journey ahead for the application of cell membrane camouflage biomimetic platforms in clinical settings. The synthesis process of membrane camouflage micro‐ and nanomaterials is intricate and inefficient, impeding their further application. Furthermore, additional confirmation is required regarding the mechanisms of action of these structural units and their specific functional proteins on the membrane. A recent research brings hope for membrane‐disguised biomimetic micro‐ and nanosystems. NV‐001 is a cancer vaccine that enhances the immunogenicity of tumor antigens by using a hybrid technique of tumor cell membranes and *Escherichia coli* bacterial membranes, thereby stimulating an immune response against cancer cells.^193^ An ongoing Phase I clinical trial aims to evaluate the safety, tolerability, preliminary efficacy, and immunogenicity of NV‐001 in the treatment of advanced solid tumors. Although patient recruitment has not yet commenced (NCT06051760), this hybrid membrane‐based cancer vaccine, if successful in clinical trials, is expected to offer new options for patients who still face the risk of tumor recurrence after surgical removal.

EVs are currently being investigated in a limited number of ongoing clinical trials, predominantly in Phase I, encompassing engineered dendritic cells, macrophages, and MSC‐derived EVs. These trials aim to augment immunotherapy or facilitate gene therapy but have yet to disclose their results. For example, a Phase II trial (NCT01159288) is assessing the efficacy of tumor antigen‐loaded dendritic cell‐derived exosomes (Dex) as an immunotherapy for NSCLC patients who are ineligible for surgical removal after completing chemotherapy. Another trial (NCT06245746) plans to evaluate the safety and efficacy of umbilical cord‐derived MSC exosomes (UCMSC‐Exo) in consolidating chemotherapy‐induced myelosuppression following complete remission in acute myeloid leukemia patients. Additionally, the macrophage reprogramming agent exoASO–STAT6 (CDK‐004) is designed to specifically deliver STAT6 antisense oligonucleotides (ASO) to myeloid cells, promoting the repolarization of macrophages from the immunosuppressive M2 phenotype to the proinflammatory M1 phenotype, thereby inducing antitumor immunity.^194^ Unfortunately, a Phase I trial (NCT05375604) assessing the safety, tolerability, pharmacodynamics, and pharmacokinetics of CDK‐004 in patients with advanced hepatocellular carcinoma and those with primary gastric cancer or CRC liver metastases has been terminated, with results not yet published, and its antitumor effects require further clinical trials for validation. Additionally, a Phase I clinical trial (NCT03608631) is underway utilizing MSC‐derived exosomes carrying siRNA targeting KRAS G12D mutation in participants with metastatic pancreatic cancer harboring the KrasG12D mutation aiming at determining optimal dosage and potential side effects. Although outcomes remain undisclosed at present time, existing clinical trials have demonstrated promising antitumor activity through KRAS G12D inhibition, which underscores the potential application of these innovative iExosomes.^195^

Cellular organelles inspired nanomaterials have achieved significant advancements in basic experimental research; however, they have not yet transitioned into clinical trials. Overcoming fabrication complexities, enhancing efficiency, and deepening the understanding of their mechanisms of action are imperative for facilitating their further translational application.

### 3.4 Challenges of the bioinspired micro‐ and nanostructured systems toward clinical translation

In the clinical translation process of bioinspired micro‐ and nanostructured systems for oncology treatment, despite demonstrating significant therapeutic potential and some having received clinical approval, various innovative therapeutic approaches still encounter persistent challenges.

#### 3.4.1 Balancing efficacy and biocompatibility

Ensuring the biocompatibility and immunocompatibility of treatments to minimize adverse reactions is a critical concern in the development of all bioinspired micro‐ and nanostructured systems. When utilizing exogenous systems such as live bacteria, viruses, or their components, it is imperative to evaluate their potential toxicity toward normal cells and tissues. To mitigate this toxicity, researchers have devised various strategies, such as utilizing nonpathogenic or attenuated microorganisms, reducing dosages, or altering surface antigens to decrease immunogenicity.^196^, ^197^ Prior to clinical application of virus‐inspired nanosystems, the possibility of other viral infections in patients must be ruled out. Consequently, further evaluation is required to assess the impact of reduced virulence on therapeutic efficacy and targeting accuracy. Similarly, comprehensive assessment is also necessary for potential immune responses that may be triggered by engineered bacteria, plants, and human cells after modification.^198^, ^199^ In summary, in the field of cancer therapy, in‐depth pharmacological and toxicological studies are still needed for biomimetic micro‐ and nanostructured systems. Additionally, as reviewed by Cabral et al.,^200^ the pharmacokinetics of nanomedicine have been emphasized as a key factor in achieving targeted drug delivery. Leveraging techniques from fields such as protein engineering, gene therapy, and regenerative medicine to enhance micro‐ and nanostructured systems represents an effective approach among many. However, unlike well‐defined synthetic materials, the complex biological structures of bioinspired micro‐ and nanostructured systems require further research and definition in terms of their pharmacodynamics and pharmacokinetics.

#### 3.4.2 Balancing individual heterogeneity and standardized quality control

Whether inspired by plants, microorganisms, or mammals, micro‐ and nanostructured systems require technological optimization during isolation, production, and drug encapsulation to enhance efficiency and purity while reducing product loss. Additionally, establishing the appropriate dosage and controlling the drug release rate are also crucial for efficacy and safety. Precise dosage control strategies are necessary for different administration routes, such as intravenous injection, intratumoral injection, or oral intake. Taking immune cells as an example, the individual differences among patients complicate quality control. Moreover, the absence of standardized and compliant production and quality control methods constitutes a significant barrier in the translation process.^201^, ^202^ As biological products, ensuring the long‐term stability of biomimetic micro‐ and nanostructured systems, as well as maintaining their biological activity and functionality during storage and transportation, is key to successful translation. Therefore, well‐designed clinical trials are needed to evaluate the safety and efficacy of biotherapeutic products and to establish standardized operating procedures and protocols for clinical application, ensuring the consistency and reproducibility of treatments, and effectively managing the risks and complexities of clinical trials.^203^

#### 3.4.3 Balancing product development with therapeutic costs and accessibility

The comprehension of the mechanisms of action of bioinspired micro‐ and nanostructured systems is still inadequate, encompassing their interactions with tumor cells, their influence on the immune microenvironment, and their in vivo behavior. For instance, fungi and organelle‐inspired micro‐ and nanostructured systems are still in the preliminary stages of fundamental research, and studies within tumor cells could facilitate the development of novel biomimetic nanocarriers. However, the high cost of therapy may restrict patient accessibility. Balancing the costs of research and development with the financial burden on patients to ensure the widespread availability and affordability of treatments is an issue that must be considered in product development.

## 4 CONCLUSIONS AND FUTURE PERSPECTIVES

Many anticancer drugs, including traditional nanomaterials, face significant limitations in penetrating physiological barriers and lack targeting capabilities, resulting in reduced therapeutic efficacy and nonspecific adverse reactions on normal cells. However, numerous biological entities found in nature demonstrate superior biocompatibility, tumor‐specific targeting abilities, and the capacity to permeate interstitial tissues when introduced into the human body. Given these limitations of current anticancer drugs in cancer therapy, bioinspired micro‐ and nanostructured systems have been developed. Sufficient evidence has proven that these plant‐, microorganisms‐, and animals‐inspired micro‐ and nanostructured systems offer significant advantages in delivering superior therapeutic outcomes while minimizing side effects due to their inherent biocompatibility, ability to penetrate biological barriers, and active targeting capabilities.

It is exciting to note that PDVs, living bacteria, bacterial components, viruses, and human cells (especially RBCs, WBCs, and MSCs or their components) have been approved for a wide range of clinical trials, and some have already been applied clinically. However, challenges persist in achieving balance between efficacy and biocompatibility, standardization and heterogeneity, as well as product development considering therapeutic costs and accessibility. In the future, hybrid nanosystems inspired by different kingdoms could be considered; for instance, integrating plant cell membranes known for their excellent biocompatibility with human tumor cell membranes possessing inherent tumor‐targeting capabilities. Naturally, prior to this advancement, it is crucial to establish large‐scale production processes is essential. Moreover, ethical considerations associated with the utilization of bio‐inspired nanomaterials in cancer treatment should not be overlooked; an example being the application of sperm robots in gynecological oncology. Lastly preclinical validation models must be developed and evaluated more comprehensively; currently immunodeficient mice are often employed to construct human tumor cell models or mouse tumor cell‐based tumor models that necessitates the creation of models that better mimic the complexities of the human body encompassing organs, tissues, and cellular levels.

## AUTHOR CONTRIBUTIONS

Yanting Shen, Tao Zhou, and Rui Yang have provided important guidance for this paper. Rui Yang, Bing Zhang, and Xiawei Fei drafted the manuscript and completed the illustrations and descriptions. Shanshan Cong and Shaojie Zhao provided the main writing ideas and further refined the article. All authors have read and approved the final manuscript.

## CONFLICT OF INTEREST STATEMENT

The authors report no conflict of interest in this work

## ETHICS STATEMENT

Not applicable.

## Data Availability

Not applicable.
